# Manipulating spatial alignment of donor and acceptor in host–guest MOF for TADF^‡^

**DOI:** 10.1093/nsr/nwab222

**Published:** 2021-12-07

**Authors:** Xiao-Ting Liu, Weijie Hua, Hong-Xiang Nie, Mingxing Chen, Ze Chang, Xian-He Bu

**Affiliations:** School of Materials Science and Engineering, TKL of Metal and Molecule-Based Material Chemistry, Nankai University, Tianjin 300350, China; MIIT Key Laboratory of Semiconductor Microstructure and Quantum Sensing, Department of Applied Physics, School of Science, Nanjing University of Science and Technology, Nanjing 210094, China; School of Materials Science and Engineering, TKL of Metal and Molecule-Based Material Chemistry, Nankai University, Tianjin 300350, China; Analytical Instrumentation Center, Peking University, Beijing 100871, China; School of Materials Science and Engineering, TKL of Metal and Molecule-Based Material Chemistry, Nankai University, Tianjin 300350, China; School of Materials Science and Engineering, TKL of Metal and Molecule-Based Material Chemistry, Nankai University, Tianjin 300350, China; State Key Laboratory of Elemento-Organic Chemistry, College of Chemistry, Nankai University, Tianjin 300071, China

**Keywords:** donor–acceptor, through-space charge transfer, thermally activated delayed fluorescence (TADF), host–guest MOF

## Abstract

Thermally activated delayed fluorescence (TADF) was achieved when electron-rich triphenylene (Tpl) donors were confined to a cage-based porous metal-organic framework (MOF) host (NKU-111) composed of electron-deficient 2,4,6-tri(pyridin-4-yl)-1,3,5-triazine (Tpt) acceptor as the ligand. The spatially separated donor and acceptor molecules in a face-to-face stacking pattern generated strong through-space charge transfer (CT) interactions with a small energy splitting between the singlet and triplet excited states (∼0.1 eV), which enabled TADF. The resulting Tpl@NKU-111 exhibited an uncommon enhanced emission intensity as the temperature increased. Extensive steady-state and time-resolved spectroscopic measurements and first-principles simulations revealed the chemical and electronic structure of this compound in both the ground and low-lying excited states. A double-channel (T_1_, T_2_) intersystem crossing mechanism with S_1_ was found and explained as single-directional CT from the degenerate HOMO−1/HOMO of the guest donor to the LUMO+1 of one of the nearest acceptors. The rigid skeleton of the compound and effective through-space CT enhanced the photoluminescence quantum yield (PLQY). A maximum PLQY of 57.36% was achieved by optimizing the Tpl loading ratio in the host framework. These results indicate the potential of the MOFs for the targeted construction and optimization of TADF materials.

## Teaser

A host-guest MOF strategy has been raised to achieve through-space charge transfer based TADF and modulable PLQY, which has been verified by spectroscopic measurements and first-principles simulations.

## INTRODUCTION

Thermally activated delayed fluorescent (TADF) materials are emerging as an important and rapidly developing research field due to their broad utility in light-emitting diodes, security protection, and fluorescence probes [1–6]. In TADF materials, triplet excitons can be efficiently utilized through reverse intersystem crossing (RISC) from a triplet excited state (T) to a singlet excited state (S); therefore, 100% internal quantum efficiency can be achieved [7,8]. From a structural design perspective, spatial separation of the highest occupied molecular orbital (HOMO) and the lowest unoccupied molecular orbital (LUMO) is needed to narrow the singlet-triplet energy splitting (Δ*E*_ST_) to promote RISC [9]. According to this principle, charge transfer (CT) interactions between donor–acceptor (D–A) molecules occur, in which the HOMO is mainly located on the donor moiety, and the LUMO is provided by the acceptor moiety [10,11]. In this way, minimal orbital overlap between the HOMO and LUMO can be achieved, which decreases Δ*E*_ST_ for effective RISC.

For TADF materials with D–A moieties, CT interactions between D and A moieties can be achieved via either through-bond or through-space mechanisms. Through-bond CT compounds require highly twisted molecular structures to achieve low orbital overlap and a small Δ*E*_ST_, which requires complicated organic design and synthesis [12,13]. Alternately, through-space CT interactions based on spatially separated donors and acceptors can effectively reduce overlap of the HOMO and LUMO by appending the orbitals to donor and acceptor moieties, respectively. Using this strategy, TADF has been reported in several classes of compounds, such as exciplexes, polymers, and organic co-crystals [14–19]. It should be noted that achieving TADF in through-space D–A compounds depends on both the characteristics of the D and A moieties and their spatial arrangements, which are related to the singlet and triplet energies and ultimately control the effective Δ*E*_ST_; therefore, achieving through-space CT-based TADF in amorphous systems is a challenging task because it is necessary to precisely control the spatial arrangements of D and A moieties [20,21]. Another challenge in TADF materials lies in the trade-off between the small Δ*E*_ST_ and high photoluminescence quantum yield (PLQY). The reduced HOMO and LUMO overlap produces a small Δ*E*_ST_ but leads to low oscillator strength, producing a low PLQY [22–24]. Overall, for TADF materials based on through-space CT interactions, controllable spatial arrangements of donors and acceptors are desired for modulating the CT strength, decreasing Δ*E*_ST_, and enhancing PLQY.

Metal-organic frameworks (MOFs) have become one of the most attractive platforms for materials development due to their highly flexible components and structural modularity [25–28]. Due to these advantages, we have previously developed a crystalline host–guest MOF for the rational construction and engineering of D–A materials [29,30]. 2,4,6-Tri(pyridin-4-yl)-1,3,5-triazine (Tpt) ligands with 1,3,5-triazine as the A moiety were interlinked by relatively strong coordination bonds with Cd^2+^ ions to form a porous host framework, NKU-111. Then, guest polyaromatic hydrocarbons as D components were incorporated into the host framework by the confined space and D–A interactions. Through-space CT interactions can be accessed using these D–A moieties. Considering the construction principle, host–guest MOFs based on D–A moieties are promising platforms for through-space CT to obtain TADF materials. First, the confined space of the host MOF framework and relatively strong coordination can fix the position and orientation of D and A molecules. Spatially separated D and A molecules can then be obtained, which leads to a small overlap of HOMO and LUMO and small Δ*E*_ST_. Proper spatial CT interactions can also be obtained, through which D and A electron clouds can interact with each other, which will enhance radiative transition processes and increase PLQY [31]. Second, the relatively rigid framework can suppress non-radiative relaxation induced by rotational and vibrational motion to further increase PLQY. In addition, the crystalline nature of MOFs allows for the straightforward structural modulation of D/A components, which is beneficial for investigating structure–property relationships.

**Scheme 1.**
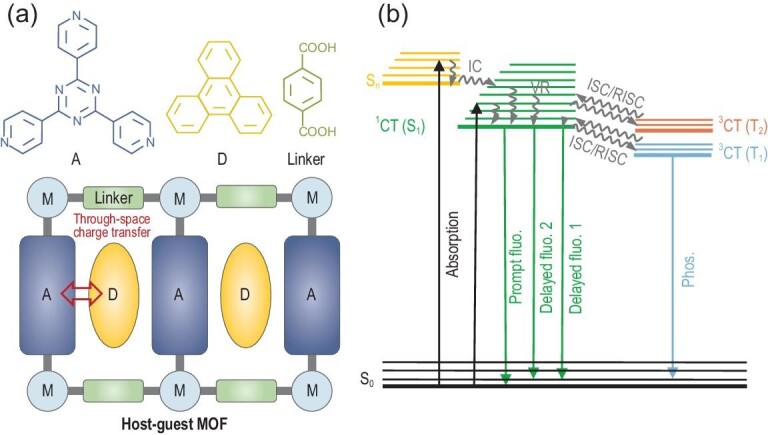
(a) Design strategy for through-space charge transfer TADF and corresponding chemical structures in the host–guest MOF Tpl@NKU-111 (M: metal center; A: acceptor; D: donor). (b) Jablonski diagram summarizing all possible fundamental photophysical processes of Tpl@NKU-111.

In this regard, here, we report achieving TADF in Tpl@NKU-111 via through-space CT (Scheme 1). By introducing electron-rich triphenylene (Tpl) molecules into the cages of NKU-111, face-to-face π–π stacking of Tpl and the electron-deficient Tpt ligand was achieved. The resulting Tpl@NKU-111 displayed enhanced emission intensity upon increasing the temperature, which fits the characteristics of TADF. Detailed photophysical characterization of Tpl@NKU-111 confirmed the presence of TADF, which was further supported by first-principles calculations. The results show that Tpl@NKU-111 had a separated LUMO and HOMO, which led to a small Δ*E*_ST_ (0.11 eV), making it an ideal platform for triplet excitation state harvesting. In addition to achieving TADF, the PLQY of Tpl@NKU-111 could be optimized by tuning the Tpl loading in the host framework. A maximum PLQY of 57.36% was achieved under an air atmosphere and room temperature when the ratio of Tpl loaded in the cages was 24.0%. These results suggest that utilizing crystalline host–guest MOFs is an effective and flexible strategy for the construction of TADF materials with intermolecular through-space CT interactions.

## RESULTS AND DISCUSSION

**Figure 1.**
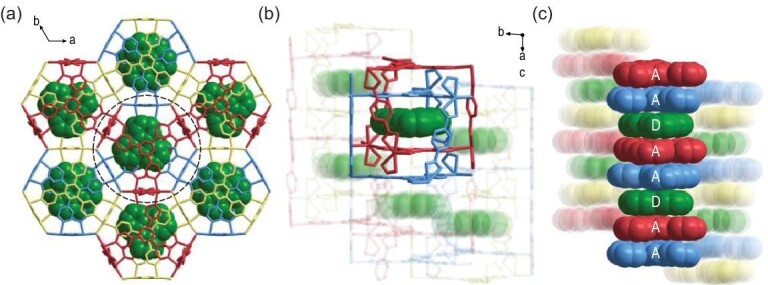
The structure of Tpl@NKU-111. (a) The three interpenetrating networks. (b) The hexagonal prism cages containing the Tpl molecules. (c) The stacking of Tpt (A) and Tpl (D).

Tpl@NKU-111 was obtained by the solvothermal assembly of Cd^2+^ ions, H_2_BDC, Tpt, and Tpl (Supplementary Fig. S1). The Tpt ligand is beneficial for enhancing D–A interactions due to its good electron-accepting ability and planar skeleton [32,33]. Conversely, electron-rich Tpl is often used as a triplet-generating material that can be assembled to display phosphorescence [34,35]. Single-crystal X-ray analysis (crystallographic data listed in Supplementary Table S1) revealed that the structure of the host framework was similar to that of pristine NKU-111, which we have reported earlier [29]. Tpl resides in the cages of the host framework in a disordered manner (only Tpl molecules at one position are shown for clarification). As shown in Fig. 1a and b, the framework was composed of three interpenetrating networks. A triangular prism cage is formed by two Tpt ligands and three BDC^2–^ ligands in the individual network. Hexagonal prism cages can then be formed by interlocking two triangular prism cages from two distinct networks. The height of the cage is about 4 Å (considering the van der Waals radius), which allows only one Tpl molecule into each cage. The confined interspace of the cage causes Tpt and Tpl to adopt a face-to-face π–π stacking mode. This guarantees that the acceptor and donor are completely spatially separated and confined in close proximity for through-space CT interactions. For the whole framework, Tpl-Tpt shows a stacking arrangement along the *c*-axis, with a DAADAA packing mode (Fig. 1c). The distances between the centers of the donors and acceptors are in the range of 3.46 to 3.58 Å (Supplementary Fig. S2), which indicates strong D–A interactions. Hexagonal prism-shaped crystals of Tpl@NKU-111 were obtained, with the preferred growth direction along the D–A stacking direction (Supplementary Fig. S3).

**Figure 2.**
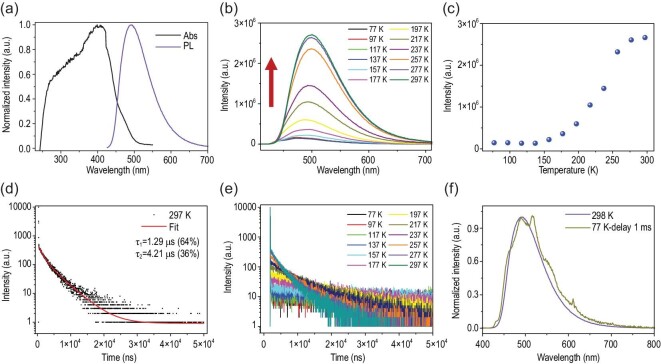
Photophysical properties of Tpl@NKU-111. (a) The absorption and PL spectra at room temperature. (b) Steady-state PL spectra at different temperatures. (c) The relationship between the PL intensity around 492 nm and temperature. (d) The PL decay curve at 492 nm and room temperature. (e) Temperature-dependent PL decay curves at 492 nm. (f) The delayed PL spectrum at 77 K and steady-state PL spectrum at 298 K.

The absorption and photoluminescence (PL) spectra of Tpl@NKU-111 were recorded to verify the occurrence of CT interactions (Supplementary Figs S4 and S5). Compared with Tpl and pristine NKU-111, the absorption spectrum of Tpl@NKU-111 shows new intense bands in the long-wavelength region around 398 nm, which is related to intermolecular CT between Tpt and Tpl molecules [36–40]. Different PL spectra were also found among the three compounds and Tpl@NKU-111 exhibited a broad, featureless emission band with peaks around 492 nm. Additionally, the large Stokes shift between the absorption and PL spectra of Tpl@NKU-111 also indicates the intermolecular CT transition (Fig. 2a). Theoretical calculations (see below) were used to simulate the absorption spectrum, and the maximum peak corresponding to S_0_–S_1_ is 394.30 nm, which is consistent with the experimental results. The maximum fluorescent peak corresponding to S_1_–S_0_ was 519.66 nm, similar to the experimental value of 492 nm. In a word, the new absorption and PL spectra were attributed to intermolecular through-space CT interactions, which can be considered as the main radiative transitions to and from S_1_.

Steady-state PL spectra of Tpl@NKU-111 at different temperatures were recorded at an excitation wavelength of 365 nm (Supplementary Fig. S6). From Fig. 2b and c, as the temperature increased from 77 K to 297 K, the PL intensity showed a nearly 20-fold increase, which is rarely observed in MOFs. At low temperatures, both TADF and phosphorescence channels are, in principle, possible. One could determine their ratios from accurate spectral simulations, for instance, in a molecular co-crystal system with a relatively simple structure [16]. While this is more challenging in a complex MOF system, we could argue the major channel is as follows. The 77 K spectrum shows double peaks, which are standard 0–0 (492 nm) and 0–1 (515 nm) vibrational transitions. The 0–0 peak coincides with the room-temperature peak; thus, it can be reasonably deduced that the major channel at low temperatures was TADF.

Thermogravimetric analysis revealed that Tpl@NKU-111 has good thermal stability, with a decomposition temperature at about 325°C, which indicates the integrity of the framework at high temperatures (Supplementary Fig. S7). Variable-temperature powder X-ray diffraction showed its non-deformable nature due to the lack of a shift or the absence of diffraction peaks (Supplementary Fig. S8). In consideration of the CT interactions responsible for emission, the uncommon positive correlation between the PL intensity and temperature might be attributed to the occurrence of TADF, in which triplet excitons were utilized by the RISC process from T_1_ to S_1_ to increase the emission intensity. To further verify the TADF feature, the PL decay curve at 492 nm was measured at room temperature. As shown in Fig. 2d and Supplementary Fig. S9, the PL decay profile exhibits a nanosecond-scale prompt component with a lifetime (τ) of 17.47 ns. Meanwhile, two microsecond-scale delayed components with τ = 1.29 μs and 4.21 μs were also observed, which indicates the co-existence of one radiative channel and two delayed radiative channels at room temperature. The temperature-dependent PL decay curves were obtained from 77 K to 297 K (Fig. 2e), and the ratio of the delayed components increased obviously upon increasing the temperature, indicating the presence of a thermally activated PL process, which is typical of TADF materials.

To determine the Δ*E*_ST_ of the system, which is a critical factor for verifying TADF, the delayed PL spectrum at 77 K and steady-state PL spectrum at 298 K were measured and compared (Fig. 2f). The former showed the maximum emission near 515 nm, and the corresponding lifetimes were 3.23 s and 0.18 s, which were attributed to phosphorescence emission originating from the lowest triplet excited state T_1_ (Supplementary Fig. S10). The steady-state PL spectrum at 298 K showed the maximum emission around 492 nm, which was assigned to fluorescence emission originating from the lowest singlet excited state S_1_. By comparing the maximum emission wavelengths of the spectra, the Δ*E*_ST_ was estimated to be 0.11 eV (S_1_, 2.52 eV; T_1_, 2.41 eV).

The photophysical properties of the physical mixture of Tpl and NKU-111 (prepared according to the component of Tpl@NKU-111), as a control, were compared. The steady-state PL spectrum at 297 K showed a series of peaks in the range of 350–450 nm, which were assigned to Tpl in consideration of the similarity of the spectrum to that of the Tpl crystals (Supplementary Fig. S12). No emission band originating from NKU-111 was found, which was attributed to the relatively low emission intensity compared with that of Tpl. The distinct emission spectrum of the mixture compared with that of Tpl@NKU-111 indicated the absence of CT interactions in the mixture, which was due to the absence of Tpl-Tpt D–A alignment. The PL decay of the mixture measured at 376 nm and room temperature was fitted by a bi-exponential function, with the main component of 0.99 ns (Supplementary Fig. S13). Meanwhile, the intensities in the decay curves decreased as the temperature increased from 77 K to 297 K (Supplementary Fig. S14). These are typical characteristics of fluorescence. In sharp contrast, new emission peaks were observed at longer wavelengths (ca. 500–600 nm) in the spectra of the mixture at 77 K (Supplementary Fig. S12). The delayed PL spectra exhibited that the intensity of the peaks decreased upon increasing the temperature, and the lifetime was determined to be 3.20 s at 496 nm and 77 K (Supplementary Figs S15 and S16). According to these results, the peaks were assigned to the phosphorescence of Tpl. These results indicated that the host–guest structure of Tpl@NKU-111 plays an important role in generating intermolecular through-space CT interactions and the corresponding TADF.

To gain deeper insights into the structure, spectra, and underlying photophysical mechanism, theoretical calculations were performed by the ONIOM (QM/MM) method with electronic embedding based on a truncated cluster model of seven hexagonal cells (2184 atoms, Supplementary Fig. S17). An A1-D-A2 fragment of one Tpl sandwiched between two Tpt moieties (with six Cd^2+^ ions bonded to them) was chosen as the high layer (108 atoms). Density functional theory (DFT) and time-dependent DFT with the Tamm-Dancoff approximation (TDDFT/TDA) were used for geometry optimizations of the ground (S_0_) and excited (S_1_, T_1_) states, respectively. The M06-2X functional was used. Extensive validations were performed for different functionals and basis sets, and stable results were achieved (Supplementary Tables S4 and S5). The low layer was modeled with a general AMBER force field.

**Figure 3.**
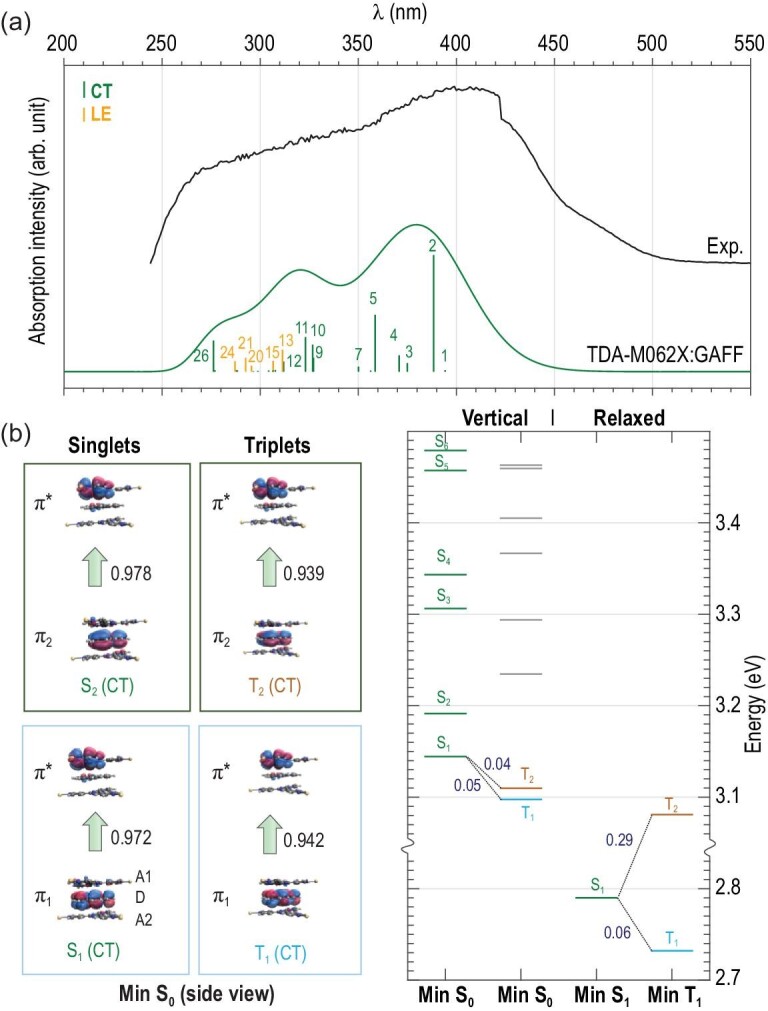
UV-Vis absorption spectra and energy levels of Tpl@NKU-111. (a) Simulated absorption spectrum compared with the experimental spectrum. The CT and LE states were characterized. (b) Computed vertical energy levels (at min S_0_) and at relaxed structures (min S_1_, min T_1_). For the two lowest singlet/triplet states (S_1_, S_2_; T_1_, T_2_) at min S_0_, state characteristics were interpreted by NTOs (bottom, hole NTO; top, particle NTO) and all were characterized as CT states (from D to A1). Occupation numbers are indicated.

Fig. 3a displays the simulated absorption spectrum in the optimized ground-state structure (min S_0_), which shows good agreement with the experimental spectrum. To understand the excited-state features of Tpl@NKU-111, we analyzed all singlet excited states within the experimental energy range in terms of the natural transition orbitals (NTOs) [41]. NTOs provide a simple single-electron picture to understand multi-electron transitions. We found that most states have evident CT features (from donor to acceptor), and only a few states at ca. 300 nm have local excitation (LE) features (localized on the donor) (Supplementary Fig. S21 and Fig. 3b). The broad peak at 398 nm (3.12 eV) in the experimental spectrum was thus characterized as a CT peak. The resolved theoretical peak was located near 380 nm (3.26 eV), which verifies the accuracy of our simulations.

The gap Δ*E*_ST_ was computed as the S_1_–T_1_ energy difference , both vertically (at min S_0_) and adiabatically (at two respectively optimized geometries min S_1_ and min T_1_). The vertical and adiabatic gaps were 0.05 eV and 0.06 eV, respectively (Fig. 3b). The theoretical adiabatic gap 0.06 eV (S_1_, 2.39 eV, 519.66 nm; T_1_, 2.33 eV, 531.29 nm) is consistent with the 0.11 eV (S_1_, 2.52 eV, 492 nm; T_1_, 2.41 eV, 515 nm) estimated from the emission experiments (summarized in Table 1). Validations with more functionals and basis sets showed similar gaps (Supplementary Table S5).

**Table 1.** A comparison of the transition energies and wavelengths for absorption (S_0_→S_1_), fluorescence (S_1_→S_0_), and phosphorescence (T_1_→S_0_) predicted from the experimental spectra and from QM/MM simulations.

Spectrum	Exp.	Theory
Abs. (S_0_→S_1_)	398 nm (3.12 eV)	394.30 nm (3.14 eV)
Fluo. (S_1_→S_0_)	492 nm (2.52 eV)	519.66 nm (2.39 eV)
Phos. (T_1_→S_0_)	515 nm (2.41 eV)	531.29 nm (2.33 eV)

In the energy level diagram (Fig. 3b), we found that the lowest two triplets, T_1_ and T_2_, have very similar energies, differing by 0.01 eV and 0.35 eV at vertical and relaxed geometries. The energy difference between T_2_ and S_1_ was also very small (0.04 and 0.29 eV, respectively); thus, besides the S_1_–T_1_ ISC/ISRC channel in normal TADF materials, we identified another channel via S_1_–T_2_. This is consistent with the two lifetimes (1.29 and 4.21 μs) obtained from experiments. NTO analysis showed that the triplets are also CT states. Although the Tpl donor has two nearest-neighbor acceptors, our analysis illustrates that only one donor (A1) is responsible for the ISC/ISRC mechanism. Structurally, this is because the two acceptors are spatially nonsymmetric relative to the donor in the vertical direction. The distances of D-A1 and D-A2 are 3.460 and 3.571 Å, respectively (Supplementary Fig. S2). In a word, the spatial difference promoted a unique CT direction within the ISC/RISC mechanism.

**Figure 4.**
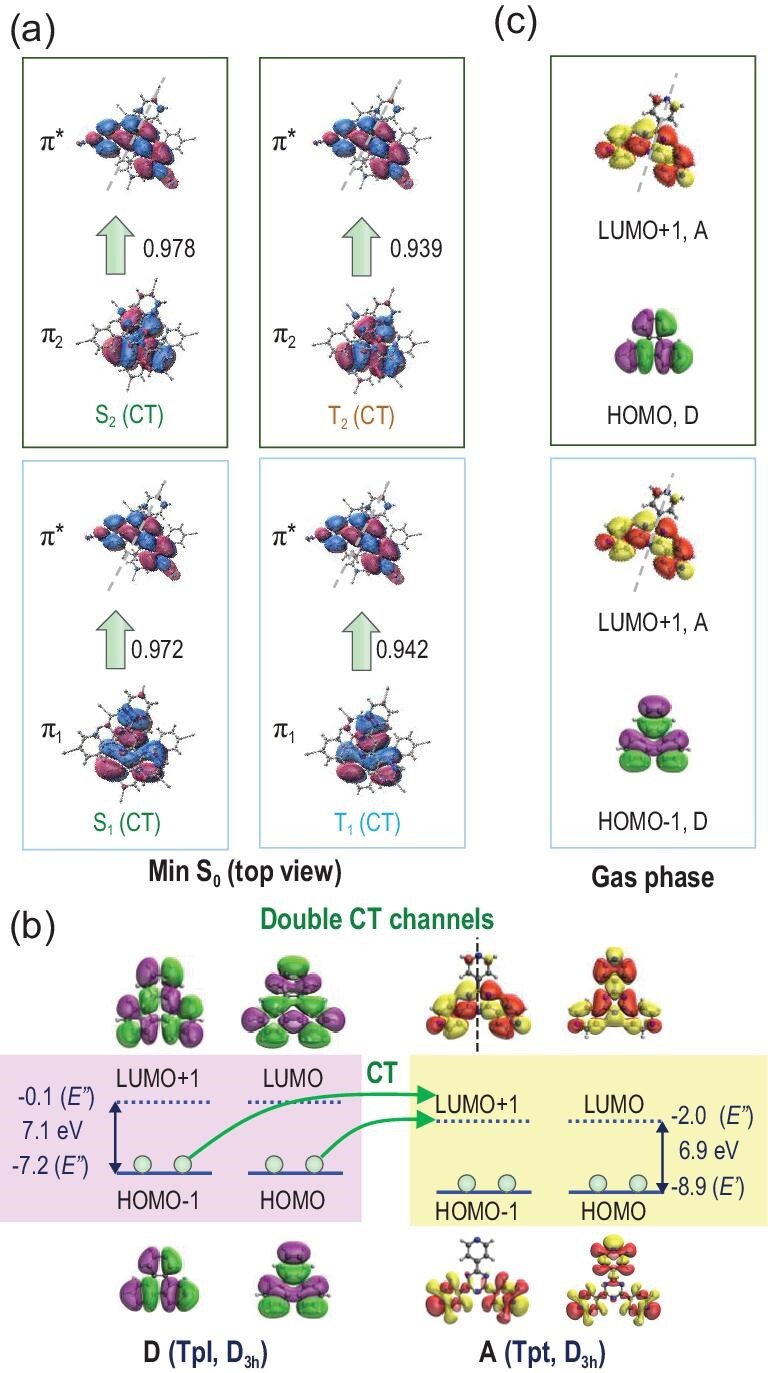
Explanation of double CT channels. (a) Top view of NTOs of min S_0_ from Fig. 3b. The major features of excited states were grouped as π_1_→π^*^ (S_1_, T_1_) and π_2_→π^*^ (S_2_, T_2_). (b) Frontier canonical orbitals, orbital energies (in eV) and symmetries, and HOMO-LUMO gaps of isolated D and A from DFT. CT from degenerate HOMO–1 and HOMO orbitals of D to LUMO+1 orbital of A is the reason for the double ISC/RISC channels in our MOF (S_1_−T_1_, S_1_−T_2_). For two degenerate orbitals, their order is not important. LUMO or LUMO+1 (HOMO−1 or HOMO) was assigned simply according to their natural order as appeared in the Gaussian output. (c) The HOMO/HOMO-1 of isolated D and LUMO+1 of isolated A (all in E’’ symmetries) show similar major characteristics of hole and particle NTOs in panel (a), respectively.

To better illustrate the D-A1 through-space CT, the top view of the NTOs is provided in Fig. 4a to better display in-plane features of the orbitals. The four excited states fall into two groups according to their characteristics: π_1_→π^*^ (S_1_ and T_1_) and π_2_→π^*^ (S_2_ and T_2_). Additional DFT calculations were performed for isolated donor and acceptor molecules, and their frontier MOs are shown in Fig. 4b. Both molecules have D_3h_ symmetry in the gas phase and have degenerate HOMO/HOMO–1 and LUMO/LUMO+1 orbital, with similar HOMO−LUMO gaps (D, 7.1 eV; A, 6.9 eV). We found that π_1_ and π_2_ are very similar to HOMO−1 and HOMO of the donor, respectively, and π^*^ shows similar character to LUMO+1 of the acceptor (Fig. 4c). Thus, the double ISC/RISC channel is better explained as arising from degenerate HOMO-1/HOMO in the donor. ISC/RISC was realized via CT from these two orbitals in the donor to the LUMO+1 orbital of the acceptor. The selectivity of LUMO+1 over LUMO in the acceptor was simply because the molecular symmetry was broken in Tpl@NKU-111, which led to energy separation. Internally, the molecule deformed from a planar structure. Externally, they are influenced by environmental groups in the cages of the host framework. For the same reason, HOMO−1 and HOMO in the donor were also separated, which led to a small energy gap between S_1_ and S_2_ (0.05 eV) and between T_1_ and T_2_ (0.01 eV). It was noticed that double-channel (T_1_−S_1_, T_2_−S_1_) intersystem crossing mechanisms were also found in single [42] or two-component [16] molecular crystals but for different reasons. The degenerate HOMO−1/HOMO in the symmetric guest donor molecule may provide a guideline for designing new MOF-type TADF materials with double-channel efficiency. The disordered Tpl in NKU-111 did not influence our analysis of the photophysical mechanisms based on our computational procedures (Supplementary Figs S18–S20); thus, our combined theoretical and experimental study validates that a good TADF material with small Δ*E*_ST_ was synthesized. These results demonstrated that the host–guest MOF is an efficient platform for TADF materials, which opens a door to TADF material design by tuning versatile D and A components in the host–guest MOF.

Besides achieving TADF, PLQY should also be considered in Tpl@NKU-111 (0.05 mmol Tpl added), which was determined to be 36.07% under an air atmosphere at room temperature. Compared with pristine NKU-111 with a PLQY of 2.41%, the higher value of Tpl@NKU-111 was attributed to the rigid skeleton of the compound, as well as proper through-space CT interactions, which stabilized triplet excitons and suppressed non-radiative transitions arising from vibrations and rotations.

**Figure 5.**
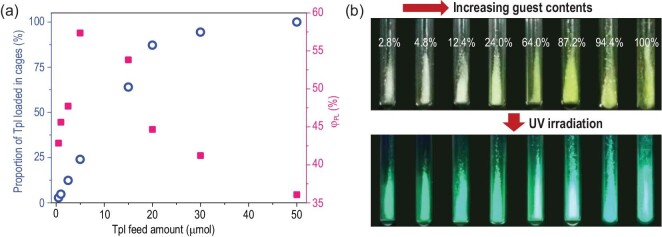
(a) Correlation between the Tpl feed amount and loading ratio in the cages of the framework (blue circle) as well as the PLQY (red square). (b) The photographs of crystal samples under daylight (top) and 365 nm UV irradiation (bottom) with different Tpl loading ratios.

Furthermore, the unique advantages of MOFs for component tuning make further PLQY optimization possible by precisely modulating the guest content. From a structural perspective, since the confined interspace of the cages in NKU-111 allows only one Tpl to be encapsulated in each cage, the loading ratio of Tpl in Tpl@NKU-111 could be tuned by adjusting the feed amount of Tpl during synthesis. To illustrate the correlation between the guest feed amount, guest loading ratio, and PLQY of the resultant compound, a series of samples was prepared with a fixed Tpt feed amount (0.05 mmol) and different Tpl feed amounts (0.5 μmol to 0.05 mmol). Powder X-ray diffraction showed the consistency of their structures (Supplementary Fig. S22). The ^1^H NMR spectra of the digested samples were obtained to determine the actual ratio of the Tpl and Tpt ligands in the different samples (Supplementary Figs S23 and S24). According to the results, the proportion of Tpl loaded in the cages in Tpl@NKU-111 could be tuned from 2.8% to 100% by changing the feed amount. A detailed analysis of the results (Fig. 5a, blue circle; Supplementary Table S2) showed a linear correlation between the Tpl feed amount and the initial cage loading. Fully-loaded cages in the framework were obtained when the Tpl feed ratio was 1.0 (0.05 mmol). The absorption and PL spectra of Tpl@NKU-111 samples with different guest feed amounts were obtained (Supplementary Figs S25 and S26). Upon increasing the guest feed amount (corresponding to increasing guest loading ratio), the absorption band near 398 nm was greatly enhanced due to intermolecular through-space CT interactions. These results fit well with the visible color change of samples upon increasing the guest loading ratio (Fig. 5b, top). In contrast, the PL spectra of the samples did not significantly change as the guest feed amount varied, which suggests that intermolecular CT was the dominant radiative channel.

The PLQY of samples with different guest contents (corresponding to the guest loading ratio) were measured at room temperature (Fig. 5a, red square) to illustrate the correlation between PLQY and guest content. At low contents, the PLQY increased to a maximum of 57.36% when increasing the guest loading ratio to 24.0%. The PLQY then decreased upon further increasing the Tpl content, which may be assigned to concentration quenching pathways [43]. A high PL intensity was achieved in the sample with a guest loading of 87.2% due to its relatively high guest content and medium PLQY. A lower PL intensity was achieved with a guest loading ratio of 100% because the PLQY decreased (Fig. 5b, bottom). Since the PL intensity of the crystalline sample was positively correlated with the guest content × PLQY, the relative PL intensity of the samples could be readily predicted (Supplementary Fig. S27). This finding is favorable for exploring the relationship between the guest content and PLQY to optimize the PL properties of this system.

## CONCLUSIONS

In summary, we have proposed a host–guest MOF strategy for through-space CT to achieve TADF with modular PLQY. This is the first time that through-space CT TADF has been achieved in MOFs. Experimental and theoretical analysis confirmed that the TADF properties arose from strong through-space intermolecular CT interactions from each donor to one of its two nearest acceptors. A double-channel (T_1_ and T_2_) intersystem crossing mechanism with S_1_ was proposed, which was attributed to CT from the degenerate HOMO−1/HOMO of the guest donor to LUMO+1 of the ligand acceptor. Based on the unique structural characteristics, the PLQY and PL intensity were precisely modulated by controlling the guest loading ratio in the cages of the MOF host structure.

Since MOFs have an ordered crystalline structure with long pathways, they provide a potential platform for investigating the structure–property relationships of TADF materials. By combining our host–guest MOF strategy and theoretical calculations, a large set of D–A components can be expected to be obtained for screening and managing the strength of D–A interactions. Subsequent optimization strategies will be disclosed, and the customization of TADF materials will be hopefully reported. We envision that this will be a significant step toward developing novel TADF materials. Multi-functionality is another important research direction, which will allow these materials to be applied in electroluminescent devices, modulable emission, organic UV photodetectors, fluorescence probes, and photocatalysis [44,45]. We will focus on developing our host–guest MOF compounds into thin-film materials to investigate the performance and the operating stability of electroluminescent devices.

## METHODS

The detailed preparation and characteristic methods of materials are available as Supplementary data.

## Supplementary Material

nwab222_Supplemental_File
