# Supplementary material for: Manipulating spatial alignment of donor and acceptor in host–guest MOF for TADF
Source: Natl Sci Rev. 2021 Dec 7;9(8):nwab222. doi: 10.1093/nsr/nwab222 (PMC9466880; doi:10.1093/nsr/nwab222)
Supplement: nwab222_Supplemental_File [file nwab222_supplemental_file.docx]

Supplementary data for

**Manipulating Spatial Alignment of Donor and Acceptor in Host-Guest MOF for TADF**

Xiao-Ting Liu^1,†^, Weijie Hua^2,†^, Hong-Xiang Nie^1^, Mingxing Chen^3^, Ze Chang^1,*^ and Xian-He Bu^1,4,*^

*^1^School of Materials Science and Engineering, TKL of Metal and Molecule-Based Material Chemistry, Nankai University, Tianjin 300350 (P. R. China)*

*^2^MIIT Key Laboratory of Semiconductor Microstructure and Quantum Sensing, Department of Applied Physics, School of Science, Nanjing University of Science and Technology, Nanjing 210094 (P. R. China)*

*^3^Analytical Instrumentation Center, Peking University, Beijing 100871 (P. R. China)*

*^4^State Key Laboratory of Elemento-Organic Chemistry, College of Chemistry, Nankai University, Tianjin 300071 (P. R. China)*

*^†^These authors contributed equally to this work.*

*Dedicated to the 100th anniversary of Chemistry at Nankai University*

^*^E-mail: changze@nankai.edu.cn (Ze, Chang); buxh@nankai.edu.cn (Xian-He, Bu).

**Section S1: Materials and Instrumentation**

All the reagents and solvents were analytical reagent (AR) grade and used without any purification, except Tpt ligand, which was synthesized according to previous report [S1].

Single crystal X-ray analysis of the **Tpl@NKU-111** crystal was performed on a Rigaku XtalAB Pro MM007 DW diffractometer Cu-Ka radiation (λ = 1.54178 Å) at 100 K. The spherical harmonics is applied to Empirical absorption correction [S2]. The structures were solved by intrinsic phasing methods using SHELXT program. Refining programs were performed using SHELXL-2018/3, and final full-matrix refinements were against F^2^ [S3]. The crystallographic data are listed in Table S1. The CCDC numbers is 2046604.

Powder X-ray diffraction (PXRD) patterns were collected on a Rigaku MiniFlex 600 diffractomer over the range from 3 to 50 ^o^ at the air atmosphere. Variable-temperature PXRD were performed on a Rigaku SmartLab diffractometer, equipped with a TTK 600 low-temperature chamber to control the temperature. A turbo vacuum pump can be further connected with the chamber to provide a vacuum atmosphere. Thermogravimetric analysis (TGA) was measured on a Rigaku Thermo Plus EVO2 8121 analyzer in air environment with a heating rate of 10 ^o^C min^-1^ from 25 to 800 ^o^C. UV-Vis spectra were collected at Persee TU-1950 UV-Vis spectrophotometer. The PL spectra of the crystal samples were performed on a Hitachi F7000 spectrophotometer. The temperature-dependent steady-state and delayed PL spectra were measured with an Edinburgh instruments FLS980 fluorescence spectrometer equipped with a R928-P PMT (200-900 nm) and Oxford Optistat DN liquid nitrogen thermostat. Temperature-dependent lifetimes in the millisecond and microsecond region were measured with the FLS980 fluorescence spectrometer and a microsecond flash-lamp (uF900) as excitation source. The absolute photoluminescence quantum yields (PLQY) were measured with an Edinburgh instruments FLS1000 fluorescence spectrometer by using an integrating sphere. ^1^H NMR spectra were measured on a Bruker 400 M spectrometer. PL images of the crystals were taken by a Nikon LV100ND equipped with a filter cube A (305−405 nm bandpass excitation filter, 400 nm dichroic mirror, and 410 nm long-pass emission filter).

**Section S2: Experimental procedure**

**Synthesis of Tpl@NKU-111.** A mixture of Cd(NO_3_)_2_•4H_2_O (30.8 mg, 0.1 mmol), H_2_BDC (16.6 mg, 0.1 mmol), Tpt (15.6 mg, 0.05 mmol), Tpl (11.4 mg, 0.05 mmol) in a 6 ml mixed solvents (DMF : H_2_O = 4 : 2) were added in a 20 ml vial. The vial was capped and heated at 92 ^o^C for one day and then hexagonal prism yellow crystals can be obtained. The samples with different Tpl loading ratio can be obtained by the similar method except for changing the Tpl feed amount (0.05 mmol) to 0.5, 1.0, 2.5, 5, 15, 20, and 30 μmol, respectively.

**^1^H NMR measurement of the digested Tpl@NKU-111 with different Tpl feed amounts.** The ratios of the Tpl and Tpt were confirmed by the ^1^H NMR measurement after digesting these crystal samples. Counting the integral area of the peaks can give an actual ratio of Tpl and Tpt molecules involved in the crystal samples. A fully-loaded cages in the framework can be obtained when the actual ratio of Tpl and Tpt is 1:2 according to the crystal structure. The procedure was as follows: The crystal samples were dissolved in a mixed solution (trifluoroacetic acid-d and dimethyl sulfoxide-d_6_). The crystals can be destroyed owing to the acid environment and the Tpl and Tpt molecules can be released and dissolved in the mixed solutions. The ^1^H NMR measurement was performed on a Bruker 400 M spectrometer and the actual ratio of Tpl and Tpt molecules can then be obtained.

**Section S3: Theoretical calculations**

**Computational methods.** All quantum chemical calculations were performed by using the hybrid ONIOM QM/MM method [S4] (QM, quantum-mechanics; MM, molecular mechanics; ONIOM, our own *n*-layered integrated molecular orbital and molecular mechanics) with electronic embedding as implemented in the Gaussian package [S5]. The QM part consists of altogether 108 atoms, including one Tpl donor (D, 30 atoms) sandwiched within two stacking Tpt acceptors (A1 and A2, each 36 atoms), as well as six Cd^2+^ ions which are bonded to nitrogen atoms in Tpt molecules (Fig. S17). It is necessary to include the metals in the QM region to properly describe the metal-organic interactions; otherwise large structural deformation happens around the Cd-N bonds during geometry optimizations. The six metal atoms were always kept frozen in all geometry optimizations, that is, only the three organic molecules were free to relax. Environmental effect was considered by including surrounding residues (Tpl, Tpt, BDC^2-^, and Cd^2+^) within a distance threshold of ξ_1_ = 13 Å to the central Tpl residue. In order to achieve neutral net charge for the entire cluster model, another slightly large distance threshold ξ_2_ = 14.63 Å was set to include a few more Cd^2+^ ions near the boundary. The entire cluster model (2184 atoms) includes interactions from the six neighboring hexagonal cells (horizontally) and the nearest stacking residues of the model on the top and bottom of the QM part (vertically). All environmental residues were kept frozen during geometrical optimizations. For the high-level method, density functional theory (DFT) and time-dependent DFT with Tamm-Dancoff approximation (TDDFT/TDA) [S6] was chosen for the ground (S_0_) and excited states (S_1_ and T_1_), respectively, where the M06-2X functional [S7] was employed. The 6-31G basis set [S8] was used for all non-metal atoms, and the LanL2DZ basis set and the LanL2DZ pseudo potential for Cd^2+^ [S9]. The general Amber force field (GAFF) [S10] was used as the low-level method. Restrained electrostatic potential (RESP) charges [S11,S12] were used for each separate residue (BDC^2-^, Tpt and Tpl) following the standard fitting procedure by using Antechamber tool [S13]. The Lennard-Jones parameters of Cd^2+^ were taken from ref. [S14]. A Gaussian lineshape with half-width-at-half-maximumum (hwhm) of 0.2 eV was used to convolute the stick absorption spectra. Graphics of molecular structures was generated by VMD [S15], and molecular orbitals by IQmol [S16] with contour isovalue = 0.02.

**Influence of structural disorder.** The SC-XRD crystal structure of **Tpl@NKU-111** contains disorder in the docking position of Tpl donor guest: within the cavity created by two Tpt acceptors A1 and A2, the donor D can locate just around the center (**Cryst-2**) or stay slightly displaced from the center (**Cryst**) (Fig. S18). To investigate the influence of structural disorder, both structures were investigated with the same computational procedure. The optimized structures of S_0_, S_1_, T_1_ states are denoted as **min S_0_, min S_1_,** **min T_1_** (from **Cryst**) and **min S_0_-2, min S_1_-2,** **min T_1_-2** (from **Cryst-2**). Each ground state structure (**min S_0_** or **min S_0_-2**) stays close to the corresponding crystal structure, with root-mean-squared distance (rmsd) of only 0.4-0.5 Å (Fig. S18 c-f). The optimized excited structures are in the vicinity of the corresponding optimized ground state structure (Fig. S18 b-e), which validated our computational settings for geometry relaxations. Absorption spectra (Fig. S19) and energy levels (Fig. S20) were computed for these two disordered structures, and both were found sensitive to structures. In Figure S19a, spectra computed at **min S_0_** exhibit the best agreement with experiment. Besides M06-2X, we additionally computed the spectra with the CAM-B3LYP functional [S17] (at the same geometry) which leads to similar results. The spectra at **min S_0_** also show evident difference with that at **Cryst**. This is simply because most low-lying states are charge transfer states (from D to A1 or A2), so that they are sensitive to the relative stacking geometry between the donor and the acceptors. Comparing spectra in panels a and b of Figure S19, results indicate that the donor is more likely to be slightly displaced from the center. Both structures were found to be close in energy: **Min S_0_-2** is 0.02 eV higher than at **min S_0_** at this level (Fig. S20c). Further validations on energies are given below. Both **minS_0_** and **min S_0_-2** exhibit small (vertical) ΔΕ_ST_ of 0.05 eV (Fig. S20 a-b). The S_1_-T_2_ difference is 0.04 and 0.02 eV, respectively, which support the two channel ISC/RISC mechanism. The excited-state energy at relaxed geometries shows evident differences in the two groups of structures. The adiabatic singlet-triplet gap ΔΕ_ST,adia_ stays at a very small value (0.06 and 0.02 eV). Electronic transitions of low-lying states at **min S_0_** and **min S_0_-2** show very similar features. Especially, the lowest lying singlets (S_1_, S_2_) and triplets (T_1_, T_2_) are all characterized as D🡪A1 transitions.

**Influence of functionals and basis sets.** To validate our calculations, single point calculations were performed at optimized geometries with the CAM-B3LYP functional [S17] and with larger 6-31G* [S8] and cc-pVDZ basis sets [S18] for non-metals. For Cd^2+^, the LanL2DZ basis set and pseudo potential was fixed; and no change was made for the low layer. In Table S3, ground state energies of **min S_0_** and **min S_0_-2** were compared. It is found that **min S_0_** stays 0.01-0.02 eV lower than **min S_0_-2** for all M06-2X results, while at the CAM-B3LYP level the order can be switched to be 0.04-0.05 eV higher. The MOF system is rather complex, and approximations were made in our calculations. For such small difference, it is thus difficult to distinguish which disorder form is more stable from energy calculations. From another point of view, the similarity in energy is consistent with the reality of disorder in guest positions from the experiment, which validates our calculations. Nevertheless, since the characters of low-lying states (S_1_, S_2_, T_1_, T_2_) and singlet-triplet gaps are essentially the same at both disorder forms, the structure disorder does not influence our analysis on photophysical mechanisms.

Since absorption spectra calculations favor **min S_0_**, more validations were only made based on the displaced structures (Tables S4-S5). Table S4 lists vertical energies at **min S_0_**. For all these levels, the predicted S_1_-T_1_ gap (ΔE_ST_) is 0.05-0.08 eV, and the S_1_-T_2_ gap is 0.03-0.05 eV. The S_2_-S_1_ and T_2_-T_1_ differences are 0.04-0.05 eV and 0.01-0.03 eV, respectively. Adiabatic values are given in Table S5, where the S_1_-T_1_ gap was predicted to be 0.03-0.06 eV. In summary, switching functional/basis set does not change our conclusions above. So for simplicity, in the main text we used only energy numbers and orbitals generated at the M062X/6-31G-LanL2DZ level in all analysis.

**Comparison with isolated molecules in gas phase.** To better understand the D-A charge transfer in our MOF material, individual Tpl and Tpt molecules (each with D_3h_ symmetry) were optimized at the M06-2X/6-31G level (in accord with the theoretical level for our MOF material) and frontier orbitals were generated in order to compare with natural transition orbitals (NTOs) of our MOF material (cluster model at geometry **min S_0_**). Their HOMO-LUMO gaps were predicted to be 7.1 and 6.9 eV, respectively. To validate the functionals and basis sets, additional single-point calculations were also carried out with the M06-2X and CAM-B3LYP functionals, each with a larger aug-cc-pVTZ basis set. As shown in Table S6, all methods predict consistent results and similar HOMO-LUMO gaps for both molecules, which are 6.9 and 7.0 eV (7.2 and 7.3 eV) by M06-2X (CAM-B3LYP).

**Section S4: Figures and Tables**


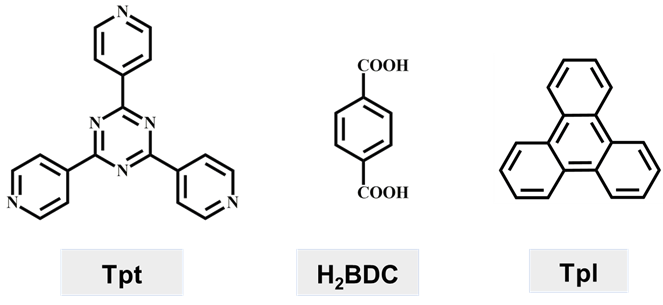


**Figure S1**. The chemical structures of Tpt, H_2_BDC and Tpl molecules.


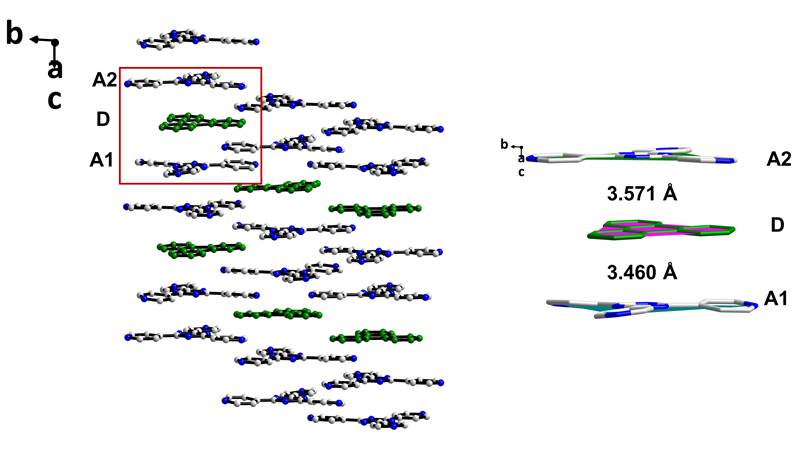


**Figure S2**. The stacking of D (Tpl) and A (Tpt) (left) and the central distances of A1-D and A2-D (right) in **Tpl@NKU-111** crystals.


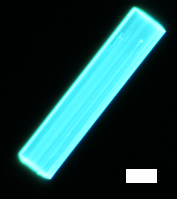


**Figure S3**. Image of **Tpl@NKU-111** crystal under UV radiation at 305-405 nm. scale bar: 25 μm.


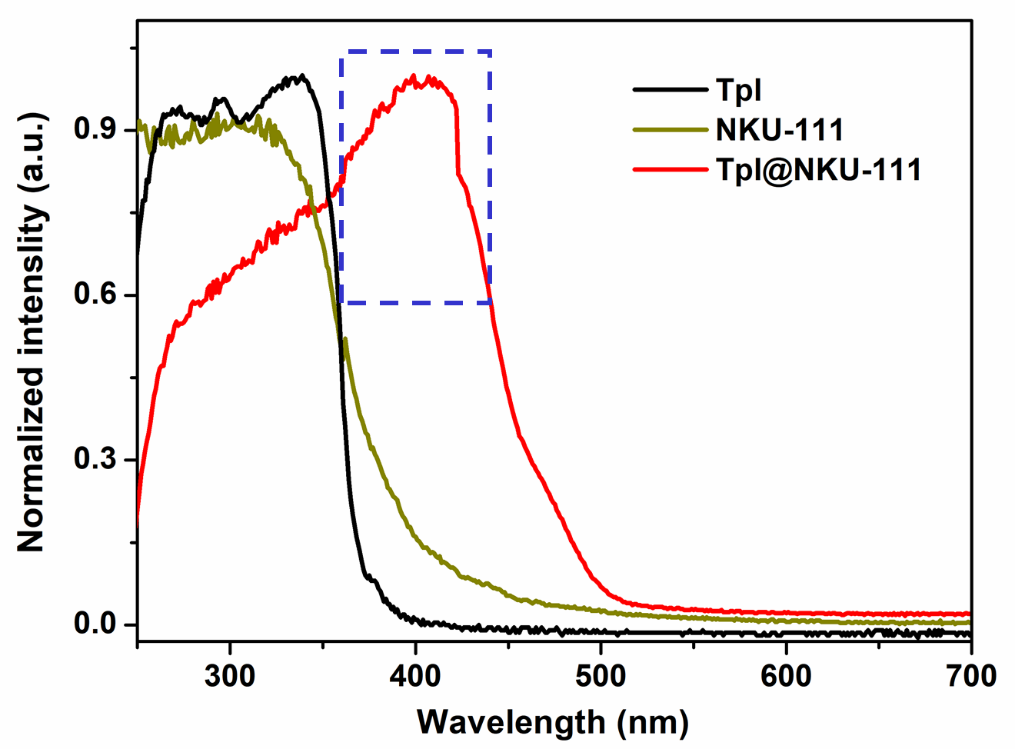


**Figure S4**. The UV-Vis absorption spectra of **Tpl@NKU-111**, pristine **NKU-111** and Tpl crystals at room temperature.


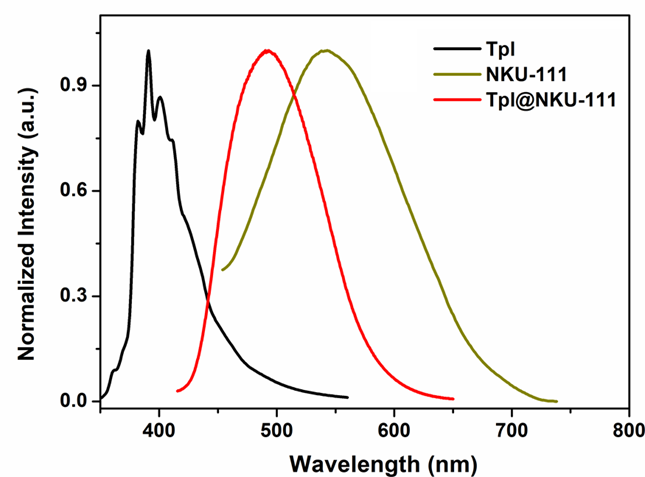


**Figure S5**. The PL spectra of **Tpl@NKU-111**, pristine **NKU-111** and Tpl crystals at room temperature.


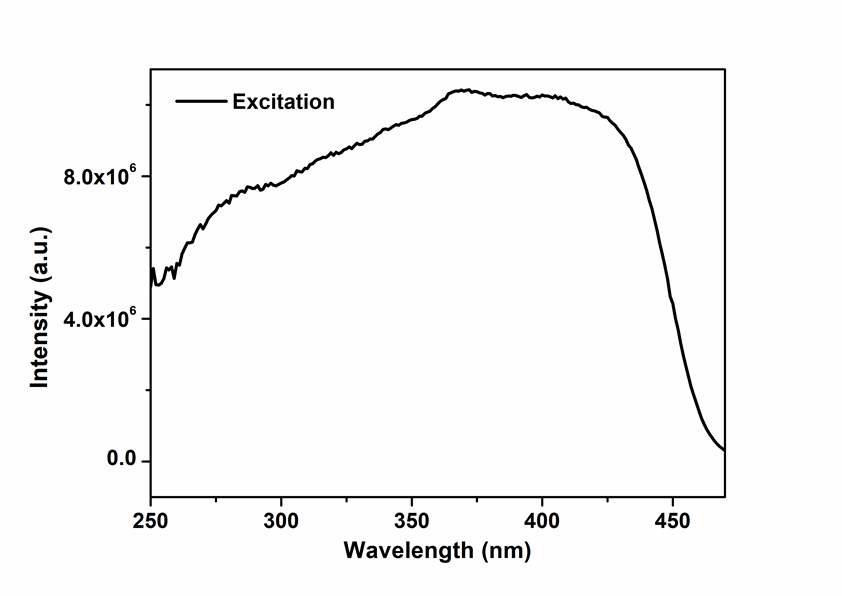


**Figure S6**. The excitation spectrum of **Tpl@NKU-111**.


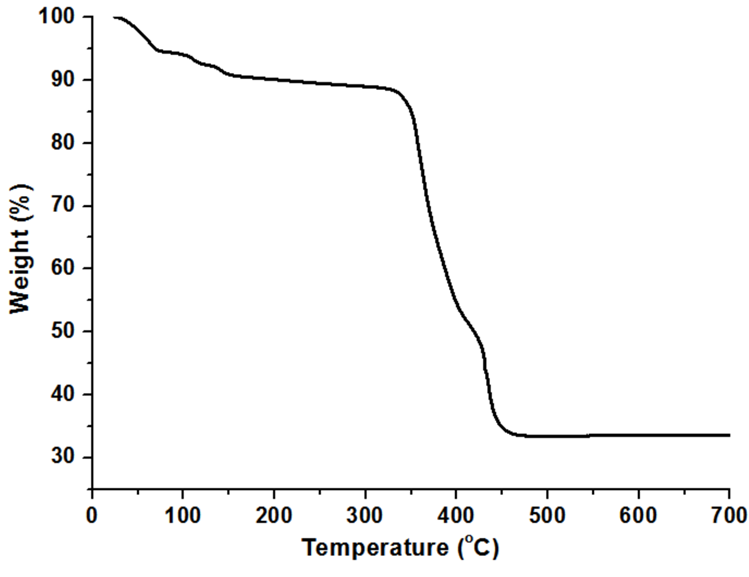


**Figure S7**. The thermogravimetric profile of **Tpl@NKU-111** at air atmosphere**.**


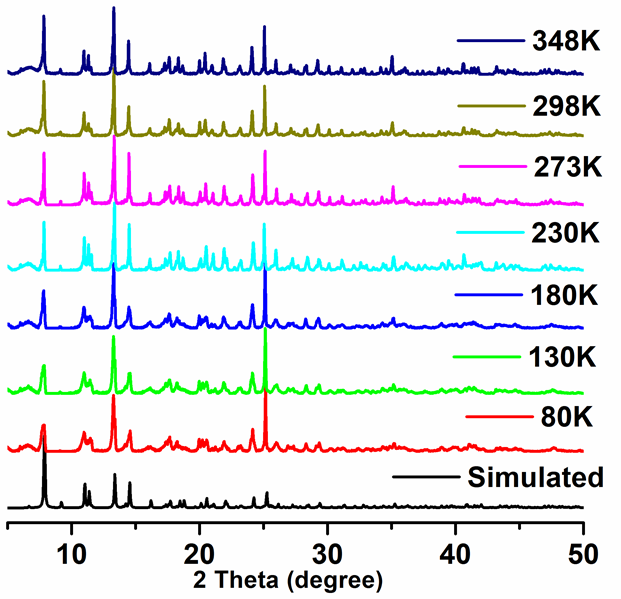


**Figure S8**. Variable temperature powder X-ray diffraction (PXRD) of **Tpl@NKU-111** at vacuum atmosphere.


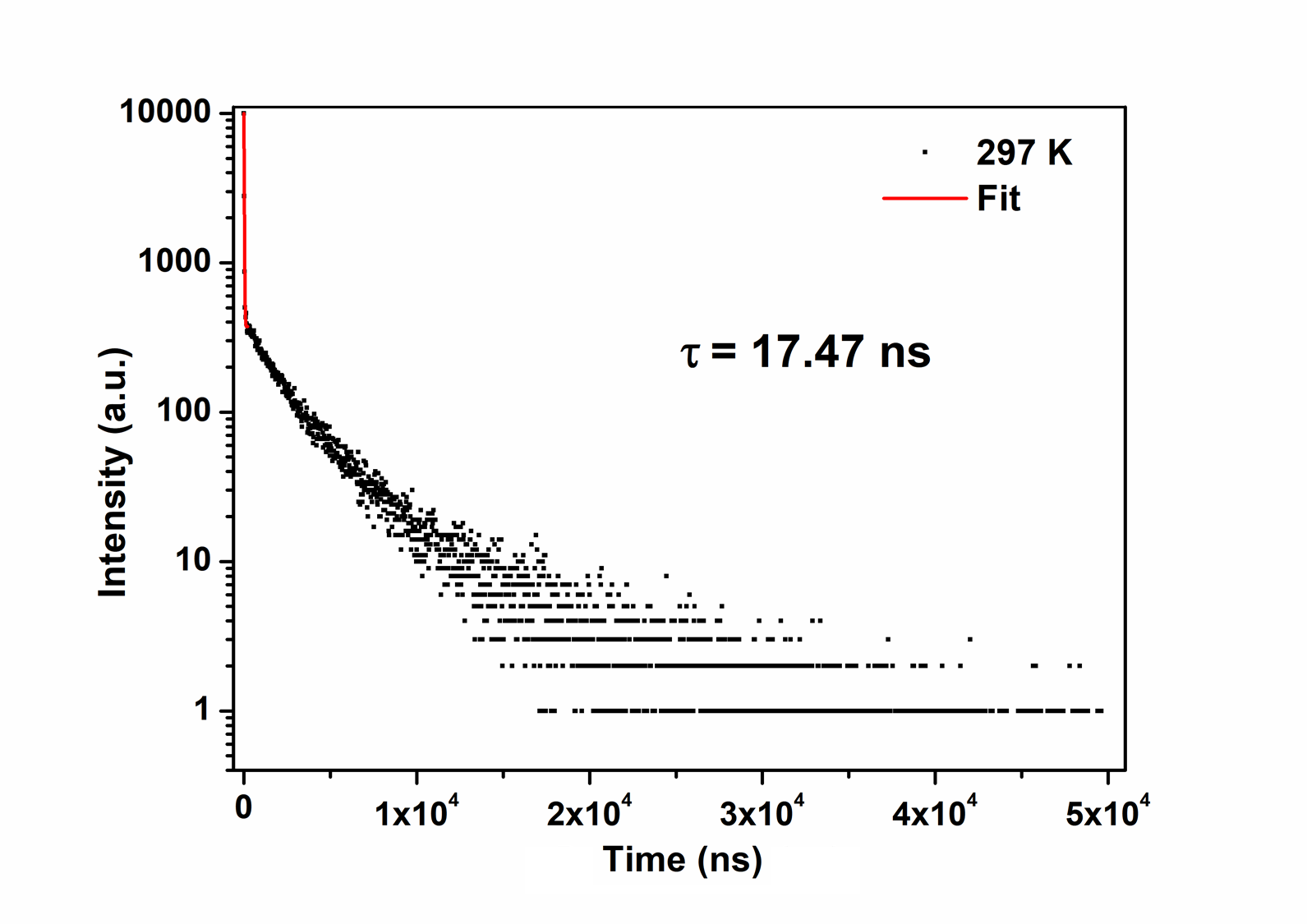


**Figure S9**. The PL decay curve of **Tpl@NKU-111** at 492 nm and room temperature.





**Figure S10**. The PL decay curve of **Tpl@NKU-111** at 515 nm and 77 K.





**Figure S11**. The powder X-ray diffraction (PXRD) of pristine **NKU-111**, Tpl and the mixture of Tpl and **NKU-111**.

PXRD pattern of the mixture is almost coincided with the superposition of the individual pattern of **NKU-111** and Tpl crystals.





**Figure S12**. The steady-state PL spectra of the mixture of Tpl and **NKU-111** at 297 K and 77 K.





**Figure S13**. The PL decay curve of the mixture of Tpl and **NKU-111** at 376 nm and room temperature.





**Figure S14**. Temperature-dependent PL decay curves of the mixture of Tpl and **NKU-111** at 376 nm.





**Figure S15**. Delayed PL spectra of the mixture of Tpl and **NKU-111** at different temperatures.





**Figure S16**. The PL decay curve of the mixture of Tpl and **NKU-111** at 496 nm and 77 K.


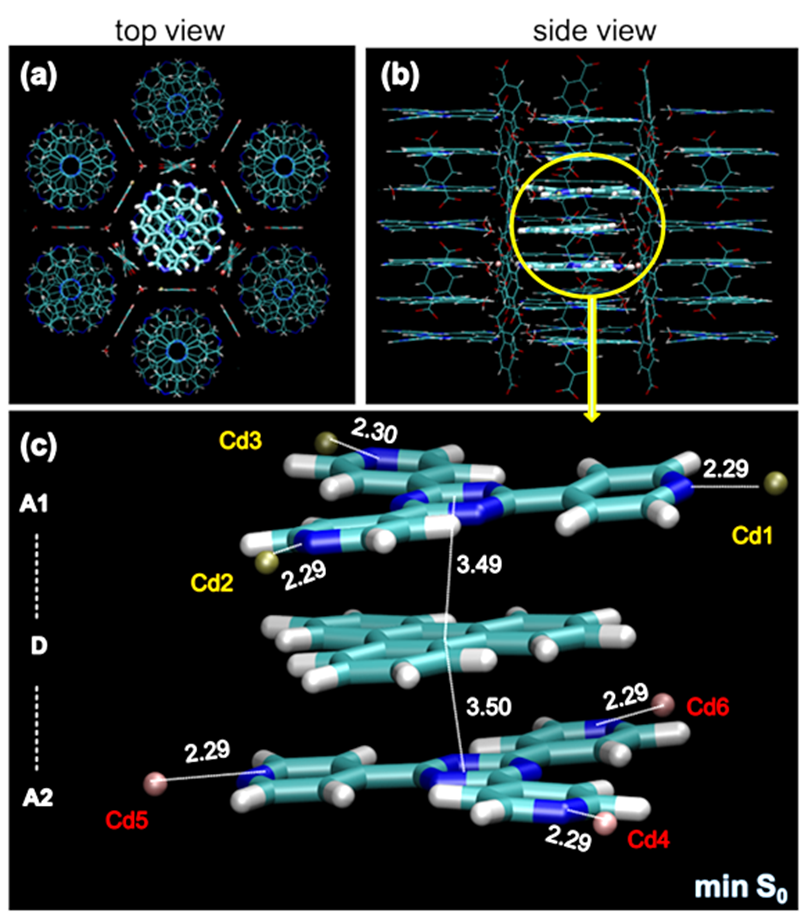


**Figure S17**. Cluster model (2184 atoms in total) used for ONIOM QM/MM simulations at the optimized geometry (**min S_0_**): (a) top view; (b) side view. The high-and low-layers are illustrated by thick tubes and thin lines, respectively; (c) Close-up view of the high layer (108 atoms). It includes a Tpl donor (D) sandwiched within two Tpt acceptors (A1 and A2), and six frozen Cd^2+^ ions. Cd atoms bonded to nitrogen atoms in A1 and A2 are distinguished by different colors (texts in gold and red, respectively). Color scheme for other atoms: C, cyan; N; blue; O, red; H, white. Cd-N bond lengths and in-plane distances are labeled (in Å).


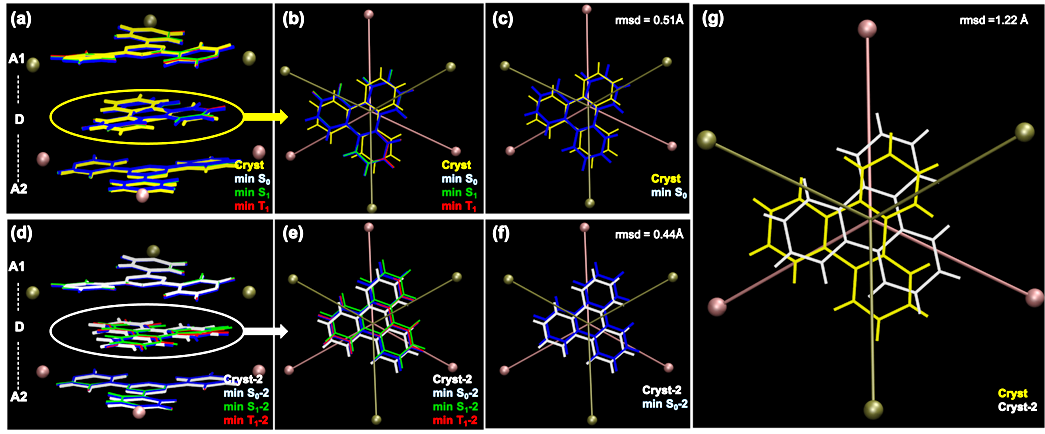


**Figure S18**. Superposition of ONIOM-optimized geometries starting from two crystal structures with disorder (**Cryst** and **Cryst-2**). Only the QM part is shown. (a-c) **Cryst** and corresponding local minima (**min S_0_, minS_1_, min T_1_**) optimized based on it; (d-f) **Cryst-2** and corresponding local minima (**min S_0_-1, minS_1_-2, min T_1_-2**). The largest structural changes lie in the relative position of the guest donor (D) within the MOF framework; (b, e) Close-up view of the donor positions: a comparison of four geometries; (c, f) Close-up view of the donor positions: a comparison of two structures (the crystal structure and corresponding optimized ground state geometry); (g) Close-up view of **Cryst** and **Cryst-2.** Selected rmsd’s of the donor geometries are labeled. In panels c-g, Cd atoms are connected with lines to guide eyes and their intersection denotes the ideal center of the cavity created between A1 and A2.


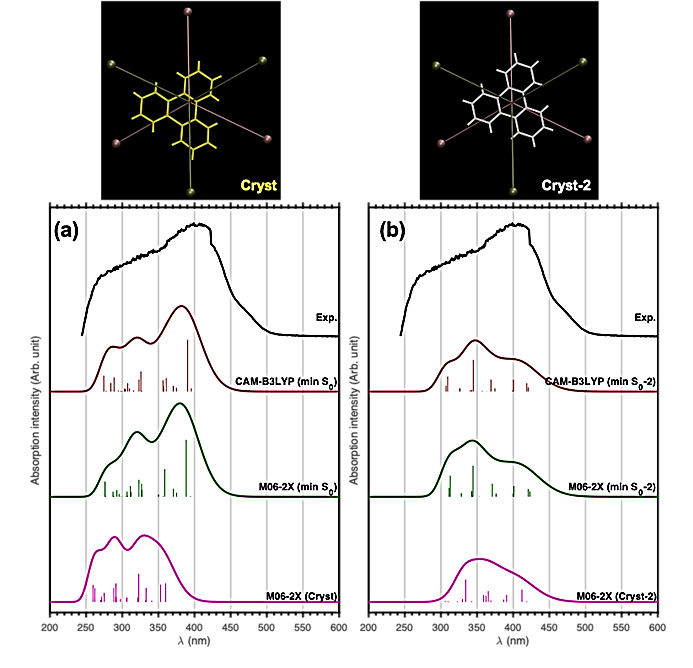


**Figure S19**. UV-Vis absorption spectra: experimental spectrum and theoretical spectra simulated at two different crystal structures with disorder (**Cryst** and **Cryst-2**) and corresponding optimized ground state geometries: (a) **Cryst** and **min S_0_**; (b) **Cryst-2** and **min S_0_-2**. Major geometrical difference of **Cryst** and **Cryst-2** lies in the donor position within the MOF cavity, as visualized on top of the figure (see also Fig. S18g). Spectra were simulated at the ONIOM (TDDFT/TDA:GAFF) level with two different functionals M06-2X and CAM-B3LYP. Theoretical spectra are compared with experimental spectrum. No *ad hoc* shift was applied.


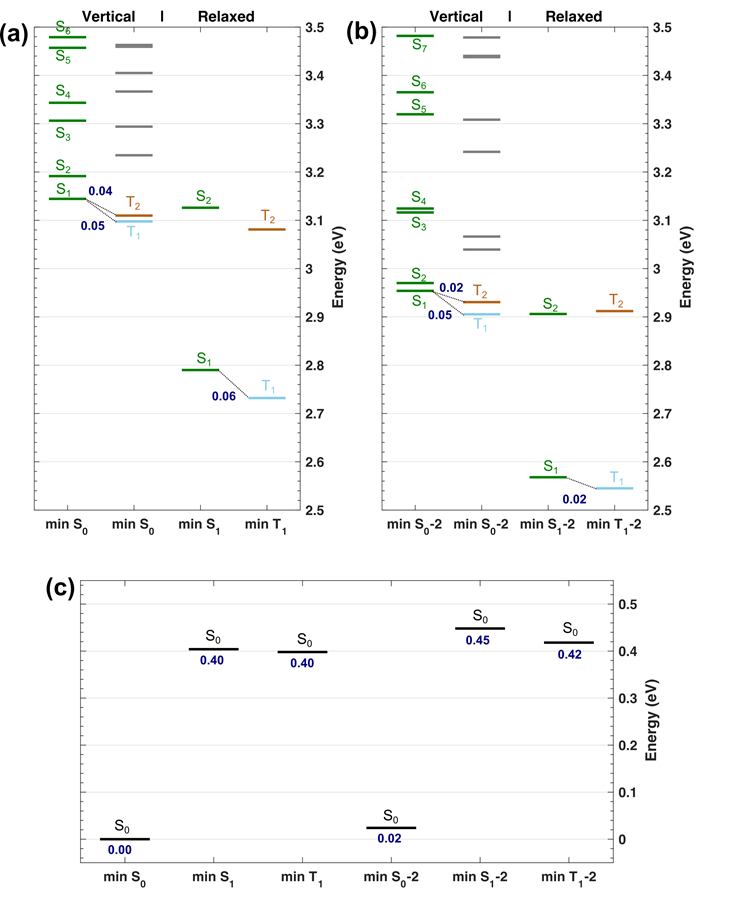


**Figure S20**. ONIOM (TDA-M062X/6-31G-LanL2DZ:GAFF) energy levels at optimized structures. (a-b) Low-lying singlet and triplet excited states at (a) **min S_0_**, **min S_1_**, **min T_1_** (the same set of data as in Fig. 3b) and (b) **min S_0_-2**, **min S_1_-2**, **min T_1_-2_._** Structures are compared in Figure S18. All low-lying singlets are as shown, T_1_ and T_2_ are all termed as charge transfer states, which are colored in green, sky blue, and brown, respectively. Other triplet states are not characterized (gray); (c) Ground state energies at six minima structures. Energy of the ground electronic state at **min S_0_** is taken as zero. Selected energies or energy gaps are explicitly labeled (in eV).

**
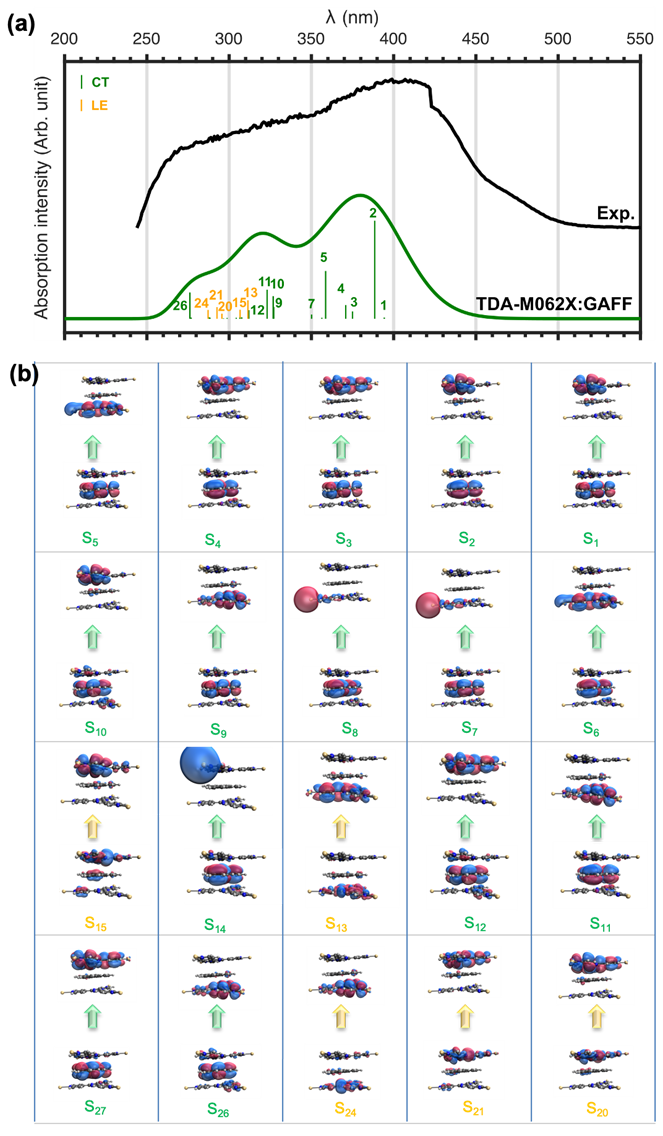
**

**Figure S21**. (a) (Recaptured from Fig. 3a in the text) Computed absorption spectra at **min S_0_** simulated at the ONIOM (TDA-M062X/6-31G-LanL2DZ:GAFF) level. Major peaks were labeled by green (charge transfer states) and gold (local excitation states on Tpl). Energies and oscillator strength values are listed in Table S7; (b) Transitions to selected S_n_ states are analyzed in terms of dominant natural transition orbitals (in each block, bottom, hole orbital; top, particle orbital). Each analyzed transition exhibits only one dominant NTO pair with the occupation number close to 1.


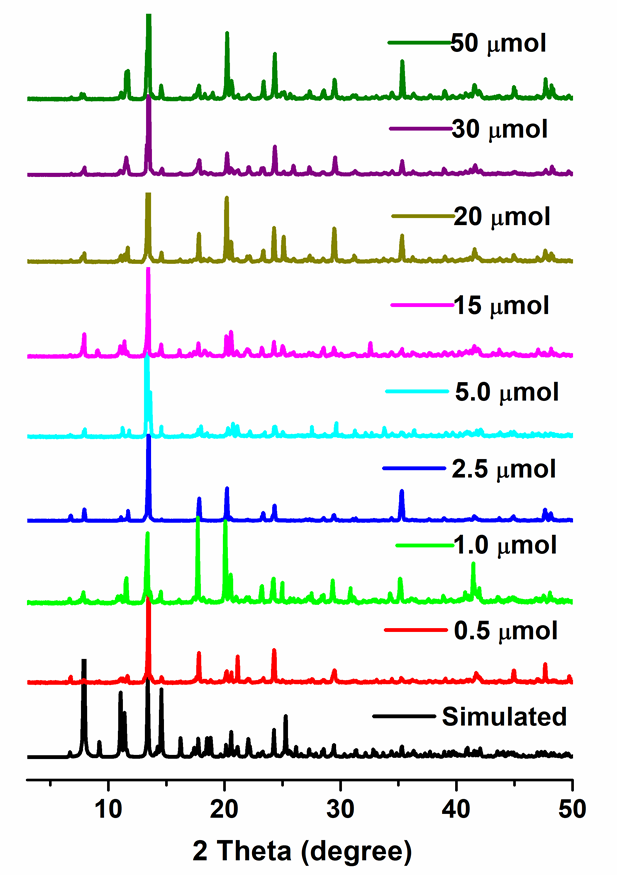


**Figure S22**. The powder X-ray diffraction (PXRD) of **Tpl@NKU-111** with different Tpl feed amount.


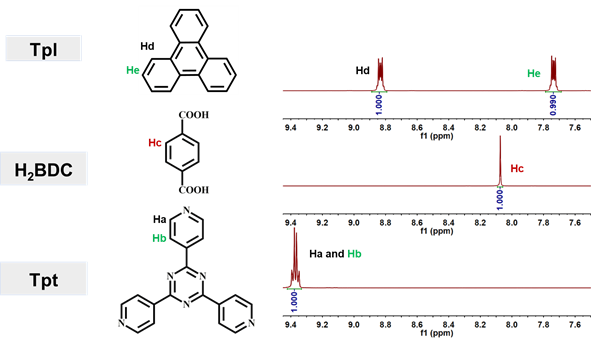


**Figure S23**. The ^1^H NMR spectra of Tpl, H_2_BDC and Tpt.


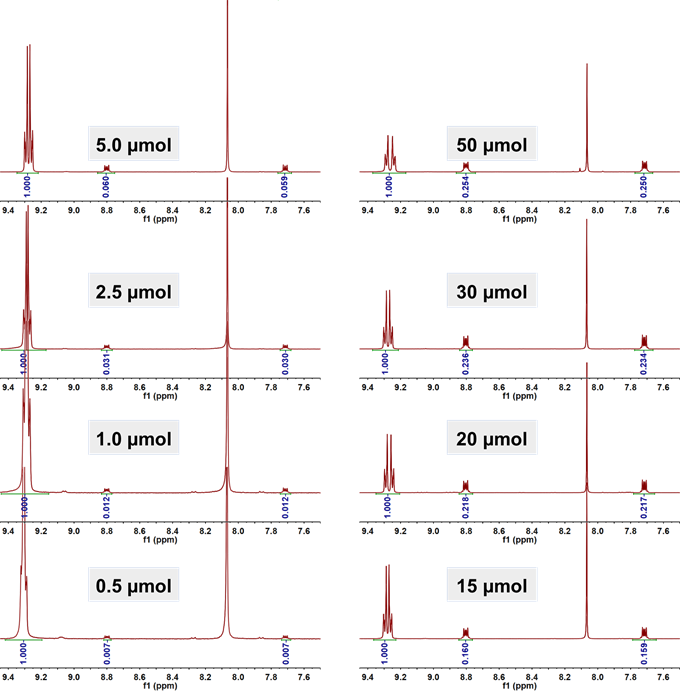


**Figure S24**. The ^1^H NMR spectra of digested **Tpl@NKU-111** crystal samples with different Tpl feed amount.


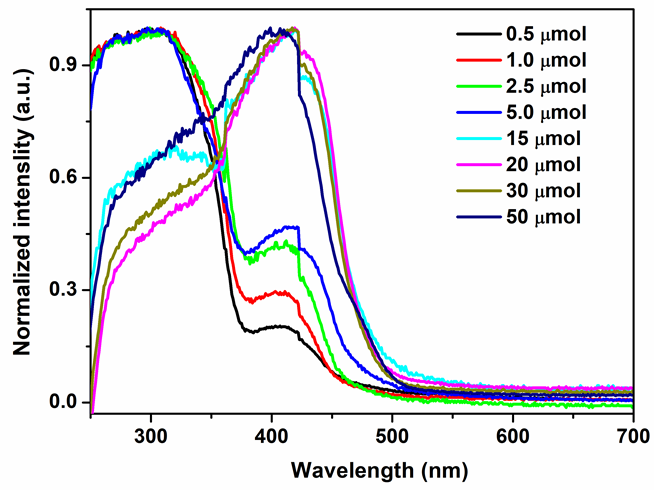


**Figure S25**. The UV-Vis absorption spectra of **Tpl@NKU-111** with different Tpl feed amount.


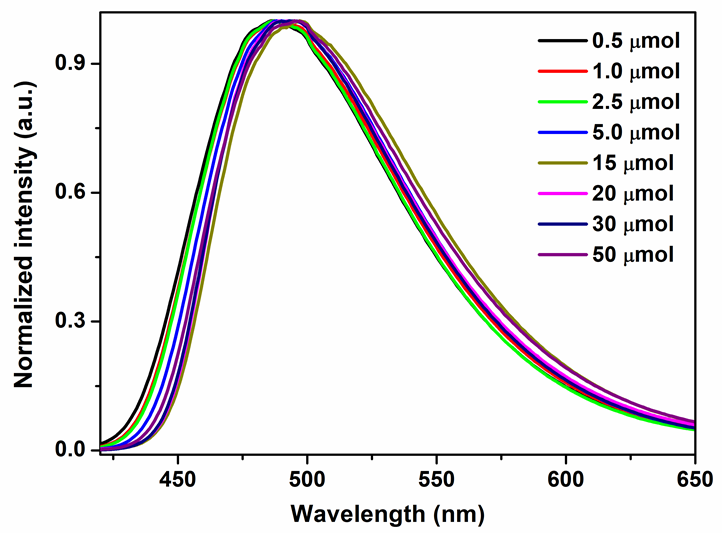


**Figure S26**. The PL spectra of **Tpl@NKU-111** with different Tpl feed amount.


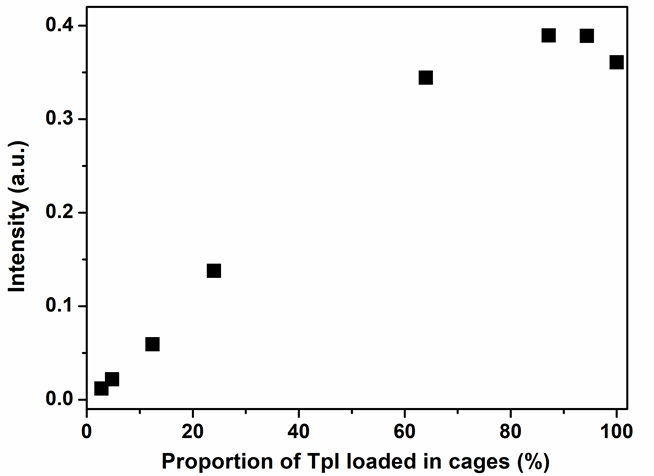


**Figure S27**. The relationship between the actual Tpl loading ratio and the theoretical emission intensity (Tpl loading × corresponding PLQY) of **Tpl@NKU-111**.

**Table S1**. Crystal structures refinement details for **Tpl@NKU-111**.

| Formula | C_78_ H_58_ Cd_3_ N_12_ O_17_ | *γ (°)* | 120 |
| --- | --- | --- | --- |
| Formula mass | 1772.56 | *V (Å^3^)* | 12814(4) |
| Temperature (K) | 100.00(10) | *Z* | 2 |
| Wavelength (Å) | 1.54178 | D_calc_ (g·m^-3^) | 1.378 |
| Crystal system | Trigonal | μ (mm^-1^) | 6.499 |
| Space group | *P*-3*c*1 | F (000) | 5340 |
| *a (Å)* | 26.454(4) | θ (º) | 3.859 to 74.553 |
| *b (Å)* | 26.454(4) | GOF | 1.066 |
| *c (Å)* | 21.144(4) | *R_1_* (I>2sigma(I))*^a^* | 0.0364 |
| *α (°)* | 90 | *wR_2_* (all data)*^b^* | 0.1155 |
| *β (°)* | 90 | CCDC | 2046604 |

^a^ *R*_1_ = Σ||*F*_o_| − |*F*_c_|| / Σ|*F*_o_|; ^b^ *wR*_2_ = [Σ[*w*(*F*_o_^2^ − *F*_c_^2^)^2^] / Σ*w*(*F*_o_^2^)^2^]^1/2^.

**Table S2**. Summary of the content of Tpl and Tpt molecules.

| Tpl feed amount (μmol) | 0.5 | 1.0 | 2.5 | 5 | 15 | 20 | 30 | 50 |
| --- | --- | --- | --- | --- | --- | --- | --- | --- |
| Feed ratio of Tpl and Tpt | 0.01 | 0.02 | 0.05 | 0.1 | 0.3 | 0.4 | 0.6 | 1.0 |
| Actual ratio of Tpl and Tpt | 0.014 | 0.024 | 0.062 | 0.12 | 0.32 | 0.436 | 0.472 | 0.508 |
| Proportion of Tpl loaded in cages (%) | 2.8 | 4.8 | 12.4 | 24.0 | 64.0 | 87.2 | 94.4 | 100 |

**Table S3**. Ground state energies of **min S_0_** and **min S_0_-2** with different functionals and basis sets (all in eV).

| Functional | Basis^a^ | E_S0_ | |
| --- | --- | --- | --- |
|  |  | **min S_0_** | **min S_0_-2** |
| M062X | 6-31G  (774) | 0.000 | 0.024 |
|  | 6-31G*  (1104) | 0.000 | 0.024 |
|  | cc-pVDZ  (1212) | 0.000 | 0.012 |
| CAM-B3LYP | 6-31G  (774) | 0.000 | -0.044 |
|  | 6-31G*  (1104) | 0.000 | -0.042 |
|  | cc-pVDZ  (1212) | 0.000 | -0.047 |

^a^ Basis set for non-metal atoms (for Cd the LanL2DZ basis set and pseudopotential was employed). Number of basis functions is included in parenthesis.

**Table S4**. Vertical transition energies for the lowest two singlet (E_S1_, E_S2_) and triplet (E_T1_, E_T2_) excited states, and their energy differences (all in eV) computed at optimized ground state geometry **min S_0_** by the ONIOM (TDA:GAFF) methods with different functionals and basis sets.^a^

| Functional | Basis^b^ | E_S1_ | E_S2_ | E_T1_ | E_T2_ | ΔE_ST_ | E_S1_-E_T2_ | E_S2_-E_S1_ | E_T2_-E_T1_ |
| --- | --- | --- | --- | --- | --- | --- | --- | --- | --- |
| M062X | 6-31G  (774) | 3.144 [394.30] | 3.192  [388.48] | 3.098  [400.26] | 3.110  [398.68] | 0.047 | 0.035 | 0.047 | 0.012 |
|  | 6-31G*  (1104) | 3.170  [391.15] | 3.222  [384.80] | 3.118  [397.67] | 3.142  [394.57] | 0.052 | 0.027 | 0.052 | 0.025 |
|  | cc-pVDZ  (1212) | 3.224  [384.53] | 3.275  [378.53] | 3.169  [391.26] | 3.195  [388.09] | 0.055 | 0.030 | 0.051 | 0.026 |
| CAM-B3LYP | 6-31G  (774) | 3.135  395.53 | 3.175  [390.50] | 3.075  [403.18] | 3.098  [400.25] | 0.059 | 0.037 | 0.040 | 0.022 |
|  | 6-31G*  (1104) | 3.153  393.17 | 3.200  [387.48] | 3.086  [401.73] | 3.119  [397.51] | 0.067 | 0.034 | 0.046 | 0.033 |
|  | cc-pVDZ  (1212) | 3.211  [386.08] | 3.256  [380.78] | 3.135  [395.51] | 3.160  [392.44] | 0.077 | 0.052 | 0.045 | 0.025 |

^a^ Computed wavelengths (in nm) included in brackets.

^b^ Basis set for non-metal atoms (for Cd the LanL2DZ basis set and pseudopotential was employed). Number of basis functions is included in parenthesis.

**Table S5**. Energies of low-lying states at optimized geometries **min S_1_** (E_S0,_ E_S1,_ E_S2_) and **min T_1_** (E_S0,_ E_T1,_ E_T2_) as well as the adiabatic S_1_-T_1_ energy difference (${\Delta E}_{\mathrm{ST}}^{\mathrm{adia}}$) computed by the ONIOM(TDA:GAFF) method with different functionals and basis sets (all in eV).^ab^

| Functional | Basis^c^ | **min S_1_** | | | | | **min T_1_** | | | | | ${\Delta E}_{\mathrm{ST}}^{\mathrm{adia}}$ |
| --- | --- | --- | --- | --- | --- | --- | --- | --- | --- | --- | --- | --- |
|  |  | $\hbar$ω_S1_ | $\hbar$ω_S2_ | E_S0_ | E_S1_ | E_S2_ | $\hbar$ω_T1_ | $\hbar$ω_T2_ | E_S0_ | E_T1_ | E_T2_ |  |
| M062X | 6-31G  (774) | 2.386  [519.66] | 2.723  [455.41] | 0.404 | 2.790 | 3.126 | 2.334  [531.29] | 2.683  [462.02] | 0.398 | 2.732 | 3.081 | 0.058 |
|  | 6-31G*  (1104) | 2.402  [516.09] | 2.746  [451.50] | 0.488 | 2.890 | 3.234 | 2.351  [527.33] | 2.703  [458.65] | 0.482 | 2.833 | 3.185 | 0.057 |
|  | cc-pVDZ  (1212) | 2.469  [502.10] | 2.811  [441.03] | 0.455 | 2.925 | 3.267 | 2.418  [512.77] | 2.766  [448.32] | 0.454 | 2.872 | 3.220 | 0.053 |
| CAM-B3LYP | 6-31G  (774) | 2.381  [520.66] | 2.722  [455.53] | 0.228 | 2.609 | 2.950 | 2.326  [533.00] | 2.680  [462.66] | 0.258 | 2.584 | 2.938 | 0.025 |
|  | 6-31G*  (1104) | 2.390  [518.78] | 2.737  [452.95] | 0.309 | 2.699 | 3.046 | 2.335  [531.08] | 2.688  [461.18] | 0.338 | 2.673 | 3.027 | 0.026 |
|  | cc-pVDZ  (1212) | 2.459  [504.18] | 2.806  [441.92] | 0.288 | 2.747 | 3.093 | 2.402  [516.23] | 2.752  [450.59] | 0.320 | 2.722 | 3.072 | 0.025 |

^a^ ${\Delta E}_{\mathrm{ST}}^{\mathrm{adia}}$= E_S1_(**min S_1_**) - E_T1_(**min T_1_**)

^b^ $\hbar$ω_X_ stands for the computed transition energies for state X, and wavelengths (in nm) are included in brackets.

^c^ Basis set for non-metal atoms (for Cd the LanL2DZ basis set and pseudopotential was employed). Number of basis functions is included in parenthesis.

**Table S6**. Computed orbitals energies (HOMO-1, HOMO, LUMO, LUMO+1) and the HOMO-LUMO gap (in eV) of the isolated Tpl donor and the Tpt acceptor in the gas phase. Different functionals and basis sets were used.

| Functional | Basis | Donor (Tpl) | | | Acceptor (Tpt) | | |
| --- | --- | --- | --- | --- | --- | --- | --- |
|  |  | HOMO-1/HOMO  (E")^a^ | LUMO/LUMO+1  (E") | gap | HOMO-1/HOMO  (E’) | LUMO/LUMO+1  (E") | gap |
| M06-2X | 6-31G | -7.15 | -0.07 | 7.09 | -8.85 | -1.99 | 6.86 |
| M06-2X | aug-cc-pVTZ | -7.40 | -0.55 | 6.84 | -9.10 | -2.13 | 6.97 |
| CAM-B3LYP | aug-cc-pVTZ | -7.45 | -0.23 | 7.22 | -9.18 | -1.86 | 7.33 |

^a^ Orbital symmetries given in parentheses.

**Table S7**. Excitation energies, wavelengths and oscillator strengths (f) at min S_0_ computed at ONIOM (TDA-M062X/6-31G-Lanl2DZ:GAFF) level.

| S_n_ | E (eV) | λ (nm) | f |
| --- | --- | --- | --- |
| 1 | 3.144 | 394.30 | 0.0007 |
| 2 | 3.192 | 388.48 | 0.0542 |
| 3 | 3.306 | 375.00 | 0.0040 |
| 4 | 3.343 | 370.84 | 0.0076 |
| 5 | 3.457 | 358.63 | 0.0264 |
| 6 | 3.479 | 356.37 | 0.0005 |
| 7 | 3.541 | 350.15 | 0.0023 |
| 8 | 3.588 | 345.52 | 0.0000 |
| 9 | 3.790 | 327.15 | 0.0065 |
| 10 | 3.794 | 326.77 | 0.0125 |
| 11 | 3.837 | 323.11 | 0.0161 |
| 12 | 3.974 | 311.96 | 0.0047 |
| 13 | 3.982 | 311.39 | 0.0102 |
| 14 | 4.030 | 307.69 | 0.0008 |
| 15 | 4.043 | 306.66 | 0.0050 |
| 16 | 4.045 | 306.53 | 0.0005 |
| 17 | 4.074 | 304.35 | 0.0005 |
| 18 | 4.150 | 298.79 | 0.0004 |
| 19 | 4.181 | 296.56 | 0.0002 |
| 20 | 4.194 | 295.62 | 0.0028 |
| 21 | 4.237 | 292.59 | 0.0064 |
| 22 | 4.300 | 288.35 | 0.0006 |
| 23 | 4.307 | 287.84 | 0.0008 |
| 24 | 4.317 | 287.20 | 0.0049 |
| 25 | 4.477 | 276.93 | 0.0007 |
| 26 | 4.489 | 276.18 | 0.0145 |
| 27 | 4.624 | 268.12 | 0.0226 |

**References**

[S1] X.-T. Liu, B. Zhao, Y.-H. Zhang, S.-S. Chen, J. Zhu, Z. Chang, X.-H. Bu, Cryst. Growth. Des. 19, 1391−1398 (2019).

[S2] Sheldrick, G. M. SADABS; Siemens Analytical X-ray Instrument Division: Madison, WI, 1995.

[S3] Sheldrick, G. M. A Short History of SHELX. Acta Crystallogr., Sect. A: Found. Crystallogr. 64, 112 (2008).

[S4] S. Dapprich, I. Komaromi, K. Byun, K. Morokuma, and M. J. Frisch, J. Mol. Struct.: THEOCHEM 461-462, 1 (1999).

[S5] M. J. Frisch, G. W. Trucks, H. B. Schlegel, G. E. Scuseria, M. A. Robb, J. R. Cheeseman, G. Scalmani, V. Barone, G. A. Petersson, H. Nakatsuji, X. Li, M. Caricato, A. V. Marenich, J. Bloino, B. G. Janesko, R. Gomperts, B. Mennucci, H. P. Hratchian, J. V. Ortiz, A. F. Izmaylov, J. L. Sonnenberg, D. WilliamsYoung, F. Ding, F. Lipparini, F. Egidi, J. Goings, B. Peng, A. Petrone, T. Henderson, D. Ranasinghe, V. G. Zakrzewski, J. Gao, N. Rega, G. Zheng, W. Liang, M. Hada, M. Ehara, K. Toyota, R. Fukuda, J. Hasegawa, M. Ishida, T. Nakajima, Y. Honda, O. Kitao, H. Nakai, T. Vreven, K. Throssell, J. A. Montgomery, Jr., J. E. Peralta, F. Ogliaro, M. J. Bearpark, J. J. Heyd, E. N. Brothers, K. N. Kudin, V. N. Staroverov, T. A. Keith, R. Kobayashi, J. Normand, K. Raghavachari, A. P. Rendell, J. C. Burant, S. S. Iyengar, J. Tomasi, M. Cossi, J. M. Millam, M. Klene, C. Adamo, R. Cammi, J. W. Ochterski, R. L. Martin, K. Morokuma, O. Farkas, J. B. Foresman, and D. J. Fox, Gaussian 09 Revision D.01 (2009), Gaussian Inc. Wallingford CT.

[S6] S. Hirata and M. Head-Gordon, Chem. Phys. Lett. 314, 291 (1999).

[S7] Y. Zhao and D. G. Truhlar, Theor. Chem. Acc. 120, 215-241 (2008).

[S8] R. Ditchfield, W. J. Hehre, and J. A. Pople, J. Chem. Phys. 54, 724 (1971).

[S9] P. Hay, W. Jeffrey, and R. Willard J. Chem. Phys. 82, 270-283 (1985)

[S10] J. Wang, R. M. Wolf, J. W. Caldwell, P. A. Kollman, and D. A. Case, J. Comput. Chem. 25, 1157 (2004).

[S11] C. I. Bayly, P. Cieplak, W. Cornell, and P. A. Kollman, J. Phys. Chem. 97, 10269 (1993).

[S12] W. D. Cornell, P. Cieplak, C. I. Bayly, and P. A. Kollman, J. Am. Chem. Soc.115, 9620 (1993).

[S13] J. Wang, W. Wang, P. A. Kollman, and D. A. Case, J. Mol. Graphics Modell. 25, 247 (2006).

[S14] Alexandre S. de Araujo, Milton T. Sonoda, Oscar E. Piro, and Eduardo E. Castellano, J. Phys. Chem. B 111, 2219-2224 (2007)

[S15] Humphrey, W., Dalke, A. and Schulten, K., "VMD - Visual Molecular Dynamics", J. Molec. Graphics 14, 33-38 (1996)

[S16] <http://iqmol.org/>.

[S17] T. Yanai, D. Tew, and N. Handy, Chem. Phys. Lett. 393, 51-57 (2004).

[S18] T. H. Dunning, J. Chem. Phys. 90, 1007–1023 (1989).
